# Twilight Sleep

**Published:** 1915-06

**Authors:** W. F. Shallenberger

**Affiliations:** Atlanta, Ga.


### Journal-Record of Medicine

Successor to Atlanta Medical and Surgical Journal, Established 1855
and Southern Medical Record, Established 1870

**OWNED BY THE ATLANTA MEDICAL JOURNAL COMPANY**

PUBLISHED MONTHLY

*Official Organ Fulton County Medical Society, State Examing Board, Atlanta, Birmingham and Atlantic Railroad Surgeon's Association, Chattahoochee Valley Medical and Surgical Association, Etc.*

RICHARD R. DALY, M. D., Editor
BERNARD WOLFF, M. D., Supervising Editor
A. W. STIRLING, M. D., C. M., D. P. H.; J. S. HURT, B. Ph., M.D.
GEO. M. NILES, M. D., W. J. LOVE, M. D., (Ala.); Associate Editors
E. W. ALLEN, Business Manager

**COLLABORATORS**

W. F. WESTMORELAND, M. D., General Surgery
F. W. McRAE, M. D., Abdominal Surgery
ALLEN H. BUNCE, Medical Societies
E. B. BLOCK, M. D., Diseases of the Nervous System
MICHAEL HOKE, M. D., Orthopedic Surgery
CYRUS W. STRICKLER, M. D., Legal Medicine and Medical Legislation
E. C. DAVIS, A. B., M. D., Obstetrics
E. G. JONES, A. B., M. D., Gynecology
ALLEN H. BUNCE, M. D., Pathology and Bacteriology
R. T. DORSEY, JR., B. S., M. D., Medicine
L. M. GAINES, A. B., M. D., Internal Medicine
GEO. C. MIZELL, M. D., Diseases of the Stomach and Intestines
L. B. CLARKE, M. D., Pediatrics
EDGAR PAULLIN, M. D., Opsonic Medicine
THEODORE TOEPEL, M. D., Mechano Therapy
R. R. DALY, M. D., Etc., Diseases of the Eye, Ear, Nose and Throat
BERNARD WOLFF, M. D., Diseases of the Skin
E. G. BALLENGER, M. D., Diseases of the Genito-Urinary Organs

VOL. LXII. ATLANTA, GA., JUNE, 1915. NO. 3

### TWILIGHT SLEEP.

W. F. SHALLENBERGER, A. M., M. D., ATLANTA, GA.

Twilight sleep or Daemmerschalaf is the term applied to that mental state produced by the administration of hyoscine or scopolamine, a mental state bordering between consciousness and unconsciousness in wihch amnesia is a promisciousness and unconsciousness in which amnesia is a prominent factor.

The production of this mental state in labor was first suggested by von Steinbuchel in 1902. Gauss and Kroenig, at Freiburg, elaborated the technic and, in 1906, published a series of 600 cases. Later other articles have appeared from time to time from the Freiburg Frauenclinic and to date they have reported over 6000 cases of labor in which Daemmerschlaf was used. Several other investigators, notably Steffens and Hoscheisen, Leopold and Veit, have written against the method as dangerous; asphyxia neonatorum, post-partum hemmorrhage and prolongation of labor were the bad results reported.

Kroenig first brought the technic of the method, as used in Freiburg, to the attention of the profession of this country at the Clinical Congress of Surgeons of North America in Chicago in Nov. 1913. Since then it has sprung into very general use largely due to the activity of the lay press and magazines.

The Freiburg technic is to give an initial dose of morphine muriate gr. 1-6 or Narcophin gr. 1-2 with Scopolamine gr. 1-150 after labor is well established and the pains are strong and regular. A second dose of scopolamine gr. 1-150 is given in about three quarters of an hour. Thereafter succeeding doses of scopolamine are guaged according a psychological index or memory tests, the idea being to keep the patient in a state of amnesia by the fewest number of doses. The patient perceives the pain but does not appperceive it and the nervous exhaustion so common after long and tedious labors or labors in women who have little or no reserve nervous force is largely done away with. It is only in a few cases that a second dose of morphine or Narcophin is given.

Siegel, at the Frieburg Clinic, undertook to standardize the dosage according to a set schedule in order to simplify the technic, but this has not been generally accepted as practicable by those men who have tried it in this country. The individualizing method seems to be the best because patients react differently to scopolamine. One thing that they insist upon at Freiburg is the use of a stable preparation of scopolamine made from the crystals either freshly prepared or rendered stable by the addition of mannite. And they use scopolaimne obtained from the scopolo plant alone and do not use hyoscine even though the identity of the two alkaloids is chemically the same. It has a sedative, narcotic effect and produces primary depression on certain of the cerebral areas. It may cause muscular incoordination, dilation of the pupil so that it will not contract during the pains, and memory is more or less obliterated. The Babinsky sign is often present when the patient is under the influence of the drug. Thirst, dryness of the throat and flushing of the face are also present to greater or less degree.

The dangers and disadvantages of the Twilight Sleep method to the mother are a possible idiosyncracy with cardiac and respiratory depression and failure; excitement instead of narcosis, rendering asepsis impossible, labor prolonged because the pains are rendered inefficient and this makes operative measures more frequent; more examinations necessary in following the progress of the labor; possibility of hemorrhage increased; mental state clouded afterward; the constant attention that must be given to the patient during the labor. Dangers to the child: Oligopnea, apnea and asphyxia; the dangers attendant upon more frequent operative measures; mentality and general condition impaired as late effects. There is not time to consider all these dangers separately. Every bad result, in cases where Twilight Sleep has been used, will naturally be assigned to its use on a post hoc basis. All these dangers and disadvantages are possible, but, in the experience of most of the men in this country, who have given the method a careful trial and have adhered to the Freiburg technic using only stable preparations of scopolamine, these dangers and disadvantages have seemed small and are outweighed by the advantages.

The statistics at Freiburg in the 6000 cases of Daemmerschlaf, as compared with a like number of cases under the older methods, show an advantage in favor of Daemmerschlaf for both mothers and babies. They get the mothers up on the third day and allow them to walk on the fourth, giving passive and active exercises on the second and third days respectively. This is neither necessary nor is it a special advantage of this method and most American writers follow their former custom in this regard. A review of the American Medical literature shows a constantly increasing number of articles on Twilight Sleep and reports of series of cases. I shall briefly enumerate a few of these investigators for I shall not have time to go into their statistics.

McPherson and Harrar at the New York Lying-In-Hospital, Rongy at the Jewish Maternity in New York, Polak at the Long Island College Hospital in Brooklyn, Beach at the Jewish Hospital in Brooklyn, Knipe at the Gouverneur Hospital in New York, Heller of New York, Libby of San Francisco, and dozen of others have given the method a thorough trial and nearly of all of them are enthusiastic in its favor in selected cases. They have all adhered to the Freibúrg technic as elaborated by Kroenig and Gauss.

Beach of Brooklyn, in the May number of the American Journal of Obstetrics, reviews the results from 1000 cass of Twilight Sleep collected from various investigators in this country, and he compares these results with those of the last 1000 cases at the Jewish Hospital in Brooklyn under the former routine. The evidence is in favor of the Twilight Sleep all through the comparison. I append charts taken from Beach's article with the warning, however, that statistics are not always reliable and one has to remember that these are hospital statistics taken from all cases in one clinic on the one hand and, in the Twilight Sleep series, they are all selected cases and one would naturally expect better results.

Baer, of Chicago, after a series of 60 cases at the Michael Reese Hospital, condemns the method in no uncertain terms. He speaks of the violence and uncertainty of it in many cases. Cragin, of the Sloane Maternity Hospital in New York, is somewhat skeptical about the advantages of Twilight Sleep and thinks it should be used with caution and only by the experienced. Bandler is also not very enthusiastic and asks:

"What do we gain?" He thinks the advantages have been exaggerated and that shock is but very little lessened by the method, and that anything that lengthens the second stage at all is a distant disadvantage. He says that Twilight Sleep has no attractions for him and that if scopolamine is used it should be given in small doses in the first stage and not at all in the second stage. Bandler claims to be able to shorten labor greatly by repeated small doses of pituitrin, using chloroform in the second stage.

My own personal experience with Twilight Sleep began last fall when I saw a few cases in Dr. Williams' Clinic at the Johns Hopkins Hospital. They were just beginning a series of cases and had only nine at that time. The cases were under the direction of Dr. Miller, one of Dr. Williams' assistants who had spent some time working in the Freiburg Clinic, and the Freiburg technic was used and the same drugs, tho they were trying to omit the last dose of scopolamine. This has been suggested by a number of the men in this country and Beach says that scopolamine should not be given within two hours of the termination of labor. All these cases at the Hopkins came thru nicely. There was no excitement and no post-partum hemorrhage. One baby was asphyxiated and would occasionally develop apnea and required watching for a number of hours and several times they had to use artificial respiration when it would stop breathing. It came around all right in the end. Labor was apparently lengthened somewhat. They were alternating normal cases that came in and thus running two parallel series, one under their regular routine and the other with the morphine-scopolamine anaesthesia. They kept close records in each series for comparison and measured the amount of blood lost. At that time Dr. Williams was not much in sympathy with the method and thought it would soon die a natural death. Since then they have used it more extensively and think a little better of it.

Since the first of the year I have used the method on nine private patients, three multiparae and six primiparae. I secured some of the same preparations that were used at Freiburg; ampules of Narcophin prepared by Boehringer and Soehne, Mannheim, and Scopolamine (Scopamannit) prepared with mannite, according to the method Straub, by Hoffman-LaRoche. Only two of these nine cases had amnesia, one complete and one partial. Three of the patients had very quick labors and received only the initial dose. Three others had comparatively easy labors and two doses were sufficient. There was some analgesia, but no amnesia in these three cases. One other patient, a primipara, after one dose of Narcophin gr. ¼ and Scopolamine gr. 1-200, became very restless and excited. The child's head was on the perineum a long time and it had apnea and required artificial respiration for twenty minutes before it would breathe naturally. The scopolamine and Narcophin were given at least three hours before the birth of the baby and ether was used during the second stage. Just be-cophin was given at least three hours before the birth of the baby and other was used during the second stage. Just before birth the heart beat of the baby dropped down well below 100 and it remained quite slow during the period of resuscitation. This was due, I think, to the long second stage and the long use of ether. I use ether in all my cases instead of chloroform and have not had any bad results, but in this case more ether was required because of the excitement of the mother. The baby has not been healthy since birth and has pylorospasm with attacks of projectile vomiting. This is much better now. It has just recovered from an attack of pneumonia when it was three and a half months old. I doubt if the one dose of Narcophin and scopolamine had anything whatever to do with the asphyxia or the poor health since birth.

In all my cases I used small amounts of ether in the second stage except one. This patient had advanced pulmonary tuberculosis and was referred to me by Dr. Paullin for he induction of premature labor. She was seven months pregnant. I gave Narcophin gr. 1-4 and scopolamine gr. 1-200 and the patient soon went to sleep and slept soundly during dilatation and packing of the cervix and the insertion of a bougie. She remained asleep for three hours. Labor pains did not begin for twelve hours and then a second dose of Narcophin gr. 1-4 and scopolamine fir. 1-400 was given. The patient slept between the pains, made no outcry and kept perfectly quiet. The birth was very easy because of the small size of the baby. It was very much emaciated and very "puny" even for a seven months baby. It cried spontaneously and breathed irregularly for a time but soon stopped and in spite of artificial respiration the heart action stopped in about half an hour. It was such a poor specimen that I was surprised to see it live that long. On the following day the woman felt better than she had for some time, and she had no recollection of anything that had occurred on the previous day after the first hypodermic injection.

It appears as tho the Twilight Sleep method of anaesthesia were here to stay, as is evidenced by the character of the men who are trying it out and advocating it in this country. It has had many criticisms, mostly from men who have not given it a fair trial and this means nothing. To paraphrase an old adage;—"The proof of the method is in the using thereof".

It undoubtedly has its dangers and disadvantages and much damage can be done by careless and injudicious use. The lay press has, so to speak, forced the hand of the doctors in many instances and men have attempted to use the method without sufficient study and experience. Many bad results have been due to the use of unstable products of scopolamine.

That Twilight Sleep is not applicable in all cases is evident. It is of chief advantage in primiparae with normal pelvic measurements. And it is not always necessary to produce a full amnesia. Small doses to produce some analgesia and the use of ether when the head reaches the perineum will help out in many cases, especially multiparae. The very fact that a woman knows that something is going to be done to help her

TABLE 1.

| | Without | With Dammerschlaf |
|---|---|---|
| Primigravidae | ...39.2% | 69.8 % |
| Multigravidae | ...60.8% | 30.2 % |
| SPONTANEOUS LABORS | | |
| Primiparae | ...73.9% | 78.36% |
| Multiparae | ...86.1% | 89.73% |
| OPERATIVE LABORS | | |
| Primiparae | ...26.1% | 20.9 % |
| Multiparae | ...13.9% | 10.27% |

TABLE II.

MATERNAL STATISTICS.

| | Without D. | With D. |
|---|---|---|
| Mortality | 1 death suddenly on 10th day, postpartum. | O. |
| Insanity | 1 melancholia, 6th day, postpartum. | [illegible] Depressive Melancholia. |
| Postpartum Hemorrhage | 17 cases, 1.7%. | 8 Cases, 0.8%. |
| Lacerations | Primiparae 91.0%<br>Multiparae 18.4%<br>Average 46.9% | 25.1%<br>3.28%<br>14.2% |
| Delirium | X. | Stable preparations, 11 cases in 546. 2% |
| | | Hyoscine and Scopolamine, 12 cases in 248, 4 8%. |

Page(s) missing

